# Comparison of coronary artery disease involvement in patients with or without familial history

**DOI:** 10.1016/j.ahjo.2025.100609

**Published:** 2025-09-10

**Authors:** Ramin Khameneh Bagheri, Hosna Hosseini Moghaddam, Mostafa Ahmadi, Ali Eshraghi, Maryam Emadzadeh, Faeze Keihanian, Aida Yavari Kondori

**Affiliations:** a Interventional Cardiologist, Department of Cardiovascular Diseases, Faculty of Medicine, Ghaem Hospital, Mashhad University of Medical Sciences, Mashhad, Iran; b Cardiology Department, Faculty of Medicine, Mashhad University of Medical Sciences, Mashhad, Iran; c Interventional Cardiologist, Department of Cardiovascular Diseases, Faculty of Medicine, Imam Reza Hospital, Mashhad University of Medical Sciences, Mashhad, Iran; d Clinical Research Development Unit, Ghaem Hospital, Mashhad University of Medical Sciences, Mashhad, Iran; e Fellowship of Echocardiography, Department of Cardiovasular diseases, Faculty of Medicine, Mashhad University of Medical Sciences, Mashhad, Iran; f Faculty of Medicine, Mashhad University of Medical Sciences, Mashhad, Iran

**Keywords:** Familial history, Coronary artery disease, Atherosclerosis

## Abstract

**Introduction:**

Family history (FH) and genetic factors contribute significantly to coronary artery disease (CAD) risk. Given limited data on this association in the Iranian population, this study assessed CAD involvement patterns among patients with and without positive familial history.

**Methods:**

This cross-sectional study was conducted from April 2019 to April 2020 at *Imam* Reza and Ghaem Hospitals, Mashhad, Iran. We enrolled 300 patients who presented with typical chest pain and confirmed CAD via coronary angiography. Participants were divided into two groups based on FH of CAD and evaluated for potential risk factors.

**Results:**

Of 300 patients, 170 had positive FH and 130 did not. The cohort included 175 males (58.3 %) with a mean age of 54.2 years. Multivariate logistic regression identified age (OR: 1.04, *p* = 0.014), male gender (OR: 0.56, *p* = 0.035), and hypertension (OR: 1.99, *p* = 0.008) as significant differentiators between FH groups. Positive FH was independently associated with more extensive CAD, including multivessel involvement and greater severity of disease.

**Conclusion:**

Positive familial history is strongly associated with increased severity and extent of CAD in Iranian patients. Incorporating FH into risk stratification can improve identification of high-risk individuals for targeted preventive and therapeutic strategies.

## 1 Introduction

Hereditary nature of coronary artery disease (CAD) is established before and this hereditary risk is influenced by a combination of genetic and environmental risk factors [1,2]. In developing countries, CAD is occurring at younger ages, affected by social and economic determinants [3,4]. Although CAD is less common among young adults, it poses significant challenges due to its impact on their active lifestyle. Risk factors, angiographic profiles, and clinical presentations differ between younger and older patients, influencing prognosis [5]. Positive familial history of CAD is strongly associated with cardiovascular disorders, independently of traditional risk factors [[6], [7], [8], [9], [10]]. Both familial history and genetic risk scores predispose individuals to acute coronary syndrome (ACS) through accelerated atherosclerosis, thrombosis, or other mechanisms. These factors have also been linked to subclinical atherosclerosis and provide additive information for predicting future cardiovascular events [11,12]. However, it remains unclear whether the increased risk in patients with a positive familial history is due to greater angiographic CAD burden or other factors [[13], [14], [15], [16], [17]].

Given the scarcity of data on this relationship in the Iranian population, this study aims to assess patterns of CAD involvement in patients with and without a positive familial history.

## 2 Methods

This cross-sectional study was conducted between April 2019 and April 2020 at *Imam* Reza and Ghaem Hospitals in Mashhad, Iran.

### 2.1 Subjects

We enrolled consecutive patients aged 18 to 65 years presenting with typical chest pain and confirmed CAD, including ST-elevation myocardial infarction (STEMI), non-STEMI, unstable angina, or chronic stable angina. Patients with incomplete clinical data were excluded.

### 2.2 Sample size

This retrospective observational study included all eligible patients meeting inclusion criteria during the defined study period. Due to the exploratory nature and data availability, no formal a priori sample size calculation was performed. Post hoc power analyses indicated that the sample size of 300 patients was sufficient to detect meaningful differences in primary outcomes.

### 2.3 Definitions

CAD was defined as ≥50 % stenosis in at least one major epicardial coronary artery. A positive family history of coronary artery disease (CAD) was defined as having a first-degree relative who developed CAD before the age of 55 years for men and before 65 years for women, according to accepted definitions in the literature. This adjustment aligns with current recommendations and captures the increased risk associated with early-onset CAD in relatives [18]. Abnormal lipid values were defined as triglycerides (TG) >150 mg/dL, low-density lipoprotein (LDL) >130 mg/dL, and high-density lipoprotein (HDL) <50 mg/dL. Angiographic patterns were classified as no significant stenosis (<50 %), left main disease (LMD), single-vessel disease (SVD), two-vessel disease (2VD), or three-vessel disease (3VD). Multi-vessel disease was defined as involvement of two or more vessels. The variable indicating prior history of ischemic heart disease (IHD) was defined based on documented clinical events including previous myocardial infarction, angina, or revascularization procedures, ascertained through detailed review of patients' medical records and clinical history interviews.

### 2.4 Study design

Patients were divided into two groups based on familial history of CAD. Risk factors (gender, diabetes mellitus, smoking, hypertension, abnormal lipid profile, and familial history) and histories of opium and smoking use (self-reported) were recorded. Coronary angiography was performed for all patients, and the angiographic patterns were compared between groups.

### 2.5 Blinding and inter-observer variability

Angiographic data were reviewed independently by two interventional cardiologists blinded to patients' familial history status. Discrepancies were resolved by consensus. Inter-observer agreement was assessed using the kappa statistic.

### 2.6 Ethics

The study was approved by the Ethics Committee of Mashhad University of Medical Sciences. Written informed consent was obtained from all participants.

### 2.7 Statistical analysis

Missing data were minimal (<5 %) for all key variables, and a complete case analysis was conducted once it was verified that the data were missing completely at random, thus minimizing the risk of bias. Continuous variables were presented as mean ± standard deviation and compared using the independent *t*-test or Mann-Whitney *U* test based on the assessment of normality via Shapiro-Wilk tests and visual inspection of histograms. Categorical variables were compared using the Chi-square test or Fisher's exact test, as appropriate. To address multiple comparisons, Bonferroni correction was applied to control for family-wise type I error. Logistic and multinomial regression models were evaluated diagnostically for multicollinearity using variance inflation factors, goodness-of-fit using the Hosmer-Lemeshow test, and for identification of influential observations; no violations of model assumptions were detected. Statistical analyses were conducted using SPSS version 19.0 (SPSS Inc., Chicago, Illinois), and a *p*-value <0.05 was considered statistically significant.

## 3 Results

This study included 300 patients with coronary artery disease confirmed by coronary angiography, comprising 170 patients with a positive family history (FH+) and 130 without (FH−). The mean age was 53.92 ± 7.07 years, with males accounting for 63 % of the cohort. Baseline characteristics are summarized in Table 1.

**Table 1:** Baseline characteristics by family history group (*N* = 300).

Variable	FH+ (*n* = 170)	FH− (*n* = 130)	p-Value
Age, years	52.4 ± 7.0	55.9 ± 6.7	<0.001
HDL (mg/dL)	39.2 ± 9.6	42.6 ± 9.6	0.003
Cholesterol (mg/dL)	194.3 ± 93.6	199.2 ± 86.0	0.642
LDL (mg/dL)	120.0 ± 71.2	120.2 ± 71.9	0.982
Triglycerides (mg/dL)	240.8 ± 143.3	238.8 ± 133.6	0.898
Fasting blood sugar	125.2 ± 65.7	114.1 ± 55.9	0.128
Sex (male), n (%)	112 (66 %)	77 (59 %)	0.237
Diabetes mellitus, n (%)	68 (40 %)	39 (30 %)	0.073
Hypertension, n (%)	101 (59 %)	60 (46 %)	0.022
Ischemic heart disease, n (%)	28 (16 %)	25 (19 %)	0.534
Hyperlipidemia, n (%)	41 (24 %)	34 (26 %)	0.687
Smoking, n (%)	63 (37 %)	51 (39 %)	0.701

Ordinal logistic regression examined associations between cardiovascular risk factors and CAD severity. Thresholds between severity categories were statistically significant (Threshold 1: B = 2.958, *p* = 0.047; Threshold 2: B = 4.474, *p* = 0.002), confirming reliable separation (Table 2).

**Table 2:** Ordinal logistic regression predicting CAD severity.

Predictor	Estimate (B)	Std. error	Wald χ^2^	p-Value	Odds ratio (Exp(B))	95 % confidence interval for OR
Age	0.029	0.017	2.888	0.089	1.030	0.996–1.064
HDL	−0.003	0.012	0.067	0.796	0.997	0.973–1.029
Cholesterol	0.005	0.003	2.855	0.091	1.005	0.999–1.010
LDL	0.000	0.003	0.001	0.970	1.000	0.994–1.005
Triglycerides	−0.002	0.001	2.107	0.147	0.998	0.995–1.001
Fasting blood sugar	0.005	0.002	4.721	0.030	1.006	1.000–1.012
Family history	0.886	0.246	13.019	<0.001	2.426	1.499–3.927
Sex	0.289	0.253	1.304	0.254	1.335	0.813–2.196
Diabetes mellitus	−0.139	0.325	0.182	0.669	0.870	0.461–1.639
Hypertension	0.319	0.249	1.645	0.200	1.376	0.844–2.243
Ischemic heart disease	−0.485	0.301	2.596	0.107	0.616	0.340–1.528
Hyperlipidemia	0.289	0.357	0.654	0.419	1.335	0.664–2.693
Smoking	−0.436	0.244	3.181	0.074	0.647	0.419–1.002

Fasting blood sugar was significantly associated with increased CAD severity (B = 0.005, SE = 0.002, *p* = 0.030), with a 0.5 % increase in odds per unit increase (OR = 1.006, 95 % CI: 1.000–1.012). Positive family history remained a strong independent predictor (B = 0.886, SE = 0.246, *p* < 0.001), with FH+ subjects having 2.43-fold higher odds of more severe CAD. Age showed a positive trend (*p* = 0.089), while HDL, cholesterol, LDL, triglycerides, sex, diabetes, hypertension, ischemic heart disease, hyperlipidemia, and smoking were not significantly associated.

Analysis of vessel involvement revealed a significant association with family history, with FH+ patients more likely to have multi-vessel disease (≥2 vessels) than FH− patients (68.2 % vs. 50.0 %, *p* = 0.001). Single-vessel disease was the most common pattern observed in FH− patients (50.0 % vs. 31.8 % in FH+). After adjusting for confounders, positive family history remained independently associated with three-vessel disease (adjusted OR = 2.22, 95 % CI: 1.20–4.06, *p* = 0.011). Age showed a borderline significant correlation, whereas history of ischemic heart disease and smoking were inversely associated (Table 3).

**Table 3:** Logistic regression analysis of factors associated with three-vessel disease.

Variable	B	Std. error	p-Value	Adjusted odds ratio (Exp(B))
FH+	0.797	0.315	0.011	2.22
Age	0.041	0.021	0.055	1.04
Sex (male)	0.124	0.323	0.701	1.13
Diabetes mellitus	−0.556	0.393	0.157	0.57
Hypertension	0.600	0.324	0.064	1.82
Ischemic heart disease	−0.802	0.362	0.027	0.45
Hyperlipidemia	0.224	0.465	0.630	1.25
Smoking	−0.800	0.306	0.009	0.45
HDL	−0.010	0.015	0.525	0.99
Cholesterol	0.000	0.004	0.978	1.00
LDL	0.002	0.003	0.572	1.00
Triglycerides	−0.002	0.002	0.403	1.00
Fasting blood sugar	0.004	0.003	0.137	1.00
Constant	−2.715	1.493	0.069	0.07

There was a significant association between family history and therapeutic recommendations (CABG, PCI, medical follow-up; Pearson χ^2^(2) = 6.224, *p* = 0.045; Table 4). After adjustment, family history was not independently associated with treatment choice. Ischemic heart disease and smoking lowered odds of medical follow-up compared to CABG, while LDL cholesterol and extent of vessel involvement predicted PCI versus CABG.

**Table 4:** Therapeutic approaches after angiography.

Treatment recommendation	Family history positive (FH+, *n* = 170)	Family history negative (FH−, *n* = 130)	Total (*n* = 300)
Coronary artery bypass grafting (CABG)	44 (25.9 %)	19 (14.6 %)	63 (21.0 %)
Percutaneous coronary intervention (PCI)	113 (66.5 %)	96 (73.8 %)	209 (69.7 %)
Medical follow-up (MFU)	13 (7.6 %)	15 (11.5 %)	28 (9.3 %)

Coronary artery dominance was significantly associated with family history status. Right dominance was more frequent in FH+ patients (90.6 %) compared to FH− patients (78.5 %, *p* = 0.003). Codominance was higher in FH− patients (6.9 % vs. 1.2 % in FH+, *p* = 0.009), while left dominance did not differ significantly (*p* = 0.080) (Table 5).

**Table 5:** Location of stenosis in coronary artery systems.

Dominance type	FH positive (FH+, n = 170)	FH negative (FH−, n = 130)	p-Value
Right dominance	154 (90.6 %)	102 (78.5 %)	0.003
Left dominance	14 (8.2 %)	19 (14.6 %)	0.080
Codominant	2 (1.2 %)	9 (6.9 %)	0.009

## 4 Discussion

Coronary artery disease is a complex disorder resulting from both inherited genetic susceptibility and environmental influences. Family history of premature CAD remains a well-established independent risk factor that likely reflects an interplay of inherited susceptibility and shared lifestyle factors within families [19,20]. Despite this, FH is not consistently incorporated in many cardiovascular risk assessment tools, which may underestimate the lifetime risk in individuals with a positive family history [21].

Our findings demonstrate that patients with a positive family history are significantly more likely to present with multivessel disease, including three-vessel involvement, compared to those without. After adjusting for conventional risk factors and demographic variables, a positive FH independently doubled the likelihood of extensive coronary artery involvement. These data underscore the role of FH as an indicator of more severe atherosclerotic burden, aligning with previous epidemiological studies linking FH to increased CAD risk and earlier onset of the disease [22,23].

Therapeutic strategies in our cohort also varied by family history status, with more invasive interventions such as coronary artery bypass grafting (CABG) and percutaneous coronary intervention (PCI) preferentially recommended in patients with FH. This likely reflects the increased anatomical severity and complexity observed in this group and highlights the clinical relevance of integrating FH data into therapeutic decision-making.

Interestingly, although lipid profiles and conventional risk factors like diabetes mellitus and hypertension were similar across groups, fasting blood sugar was independently associated with CAD severity, suggesting that glycemic control remains a critical pathway in disease progression, particularly in genetically predisposed individuals.

Our study aligns with recently published data indicating that FH improves risk prediction over conventional clinical factors and may identify individuals at heightened risk for severe CAD and adverse outcomes [24,25]. Incorporating FH into risk prediction models and anatomical scoring systems, such as the SYNTAX score, could enhance prognostic accuracy and stratify patients more effectively for precise treatment strategies [26].

Emerging technologies like artificial intelligence and machine learning are promising adjuncts for CAD risk assessment. Studies demonstrate that AI algorithms analyzing electrocardiographic features and other clinical data can improve early detection and risk stratification of obstructive CAD [27,28]. Integrating FH into these advanced prediction tools could further personalize patient care and optimize resource utilization.

Nonetheless, this study has limitations. Family history data were obtained via self-reporting, which may introduce recall bias and misclassification. Confirmation through medical records would strengthen validity but is often impractical in routine settings. Additionally, since participants were recruited from patients undergoing coronary angiography for suspected CAD, findings may not generalize to asymptomatic populations or primary prevention cohorts. Residual confounding and selection bias are inherent challenges in observational research and should be considered when interpreting the results.

## 5 Conclusion

In conclusion, positive family history of premature CAD significantly associates with more extensive and severe coronary involvement and influences therapeutic decisions. Incorporation of family history into clinical assessment and risk prediction models is essential for better identification of high-risk patients who might benefit from aggressive management and preventive strategies.

## CRediT authorship contribution statement

**Ramin Khameneh Bagheri:** Writing – review & editing, Writing – original draft, Supervision, Investigation, Data curation, Conceptualization. **Hosna Hosseini Moghaddam:** Writing – review & editing, Investigation, Data curation. **Mostafa Ahmadi:** Validation, Project administration, Data curation. **Ali Eshraghi:** Resources, Investigation. **Maryam Emadzadeh:** Formal analysis. **Faeze Keihanian:** Writing – review & editing, Writing – original draft, Visualization, Data curation. **Aida Yavari Kondori:** Writing – original draft, Data curation.

## Ethical statement

The informed consent was obtained for experimentation with human subjects. This study carried out in accordance with The Code of Ethics of the World Medical Association (Declaration of Helsinki) for experiments involving humans.

## Declaration of competing interest

There is no conflict of interest.

## References

[1] Fischer M., Mayer B., Baessler A., Riegger G., Erdmann J., Hengstenberg C., Schunkert H. (2007). Familial aggregation of left main coronary artery disease and future risk of coronary events in asymptomatic siblings of affected patients. Eur. Heart J..

[2] Keihanian F., Bigdelu L. (2020). Cardiovascular considerations in COVID19: a comprehensive review. Ther. Clin. Risk Manag..

[3] Moshar S., Najibpour R., Mohsenikia M., Bayesh S., Rad K.V., Rahimi N. (2016). Comparison of angiography findings in Iranian patients younger and older than 50 years underwent coronary angiography in boo-Ali hospital: a cross-sectional study. Thrita.

[4] Ghaderi F., Samim H., Keihanian F., Danesh Sani S.A. (2018). The predictive role of aortic propagation velocity for coronary artery disease. BMC Cardiovasc. Disord..

[5] Hassan Z., Farooq S., Nazir N., Iqbal K. (2014). Coronary artery disease in young: a study of risk factors and angiographic characterization in the Valley of Kashmir. Int. J. Sci. Res. Publ..

[6] Lloyd-Jones D.M., Nam B.-H., D’Agostino R.B., Levy D., Murabito J.M., Wang T.J. (2004). Parental cardiovascular disease as a risk factor for cardiovascular disease in middle-aged adults: a prospective study of parents and offspring. JAMA.

[7] Myers R.H., Kiely D.K., Cupples L.A., Kannel W.B. (1990). Parental history is an independent risk factor for coronary artery disease: the Framingham Study. Am. Heart J..

[8] Scheuner M.T., Setodji C.M., Pankow J.S., Blumenthal R.S., Keeler E. (2010). General Cardiovascular Risk Profile identifies advanced coronary artery calcium and is improved by family history: the multiethnic study of atherosclerosis. Circ. Cardiovasc. Genet..

[9] Poorzand H., Fazeli B., Khajavi O., Gholoobi A., Keihanian F., Morovatdar N. (2023). Association of polymorphisms of renin angiotensin system and endothelial nitric oxide synthase genes with premature cardiovascular disease in an Iranian population. BMC Cardiovasc. Disord..

[10] Poorzand H., Fazeli B., Khajavi O., Gholoobi A., Keihanian F., Morovatdar N. (2023). Association of polymorphisms of renin angiotensin system and endothelial nitric oxide synthase genes with premature cardiovascular disease in an Iranian population. BMC Cardiovasc. Disord..

[11] Tada H., Melander O., Louie J.Z., Catanese J.J., Rowland C.M., Devlin J.J. (2016). Risk prediction by genetic risk scores for coronary heart disease is independent of self-reported family history. Eur. Heart J..

[12] Keihanian F., Poorzand H., Saeidinia A., Eshraghi A. (2022). Echocardiographic and electrocardiographic findings in COVID-19 patients: a cross-sectional study. Int. J. Card. Imaging.

[13] Nasir K., Michos E.D., Rumberger J.A., Braunstein J.B., Post W.S., Budoff M.J., Blumenthal R.S. (2004). Coronary artery calcification and family history of premature coronary heart disease: sibling history is more strongly associated than parental history. Circulation.

[14] Taraboanta C., Wu E., Lear S., DiPalma S., Hill J., Mancini G.J., Frohlich J. (2008). Subclinical atherosclerosis in subjects with family history of premature coronary artery disease. Am. Heart J..

[15] Thanassoulis G., Peloso G.M., Pencina M.J., Hoffmann U., Fox C.S., Cupples L.A. (2012). A genetic risk score is associated with incident cardiovascular disease and coronary artery calcium: the Framingham Heart Study. Circ. Cardiovasc. Genet..

[16] Bigdelu L., Alimi H., Fazlinezhad A., Ghaderi F., Poorzand H., Vakilian F. (2024). Novel echocardiographic parameters in pulmonary hypertension assessment and the relationship between echocardiography and 6-minute walking test. Medicine (Baltimore).

[17] Ahmadi M., Bagheri R.K., Keihanian F., Saeidinia A. (2018). Comparison of fluoroscopy time in different catheter-engagement approaches to graft vessels in post-coronary artery-bypass graft angiography. Res. Rep. Clin. Cardiol..

[18] Khera A.V., Kathiresan S. (2017). Genetics of coronary artery disease: discovery, biology and clinical translation. Nat. Rev. Genet..

[19] Friedlander Y., Kark J., Stein Y. (1985). Family history of myocardial infarction as an independent risk factor for coronary heart disease. Heart.

[20] Hopkins P.N., Williams R.R., Kuida H., Stults B.M., Hunt S.C., Barlow G.K., Ash K.O. (1988). Family history as an independent risk factor for incident coronary artery disease in a high-risk cohort in Utah. Am. J. Cardiol..

[21] Grundy S.M., Pasternak R., Greenland P., Smith S., Fuster V. (1999). AHA/ACC scientific statement: assessment of cardiovascular risk by use of multiple-risk-factor assessment equations: a statement for healthcare professionals from the American Heart Association and the American College of Cardiology. J. Am. Coll. Cardiol..

[22] Lloyd-Jones D.M., Nam B.H., D’Agostino R.B., Levy D., Murabito J.M., Wang T.J. (2004). Parental cardiovascular disease as a risk factor for cardiovascular disease in middle-aged adults: a prospective study of parents and offspring. JAMA.

[23] Michos E.D., Vasamreddy C.R., Becker D.M., Yanek L.R., Moy T.F., Fishman E.K. (2005). Women with a low Framingham risk score and a family history of premature coronary heart disease have a high prevalence of subclinical coronary atherosclerosis. Am. Heart J..

[24] Thanassoulis G., Peloso G.M., Pencina M.J., Hoffmann U., Fox C.S., Cupples L.A. (2012). A genetic risk score is associated with incident cardiovascular disease and coronary artery calcium: the Framingham Heart Study. Circ. Cardiovasc. Genet..

[25] Khera A.V., Kathiresan S. (2017). Genetics of coronary artery disease: discovery, biology and clinical translation. Nat. Rev. Genet..

[26] Hayıroğlu M., Keskin M., Uzun A.O., Bozbeyoğlu E., Yıldırımtürk Ö., Kozan Ö., Pehlivanoğlu S. (2018). Predictive value of SYNTAX score II for clinical outcomes in cardiogenic shock underwent primary percutaneous coronary intervention; a pilot study. Int. J. Card. Imaging.

[27] Yilmaz A., Hayıroğlu M., Salturk S., Pay L., Demircali A.A., Coşkun C. (2023). Machine learning approach on high risk treadmill exercise test to predict obstructive coronary artery disease by using P, QRS, and T waves’ features. Curr. Probl. Cardiol..

[28] Cicek V., Orhan A.L., Saylik F., Sharma V., Tur Y., Erdem A. (2025). Predicting short-term mortality in patients with acute pulmonary embolism with deep learning. Circ. J..

